# An outbreak of influenza A(H1N1)pdm09 antigenic variants exhibiting cross-resistance to oseltamivir and peramivir in an elementary school in Japan, September 2024

**DOI:** 10.2807/1560-7917.ES.2024.29.50.2400786

**Published:** 2024-12-12

**Authors:** Emi Takashita, Kohei Shimizu, Shuzo Usuku, Ryuichi Senda, Ichiro Okubo, Hiroko Morita, Shiho Nagata, Seiichiro Fujisaki, Hideka Miura, Noriko Kishida, Kazuya Nakamura, Masayuki Shirakura, Masataka Ichikawa, Yoko Matsuzaki, Shinji Watanabe, Yoshimasa Takahashi, Hideki Hasegawa

**Affiliations:** 1 Research Center for Influenza and Respiratory Viruses, National Institute of Infectious Diseases, Tokyo, Japan; 2 Yokohama City Institute of Public Health, Kanagawa, Japan; 3 Ichikawa Children’s Clinic, Kanagawa, Japan; 4 Yamagata University Faculty of Medicine, Yamagata, Japan; 5 Research Center for Drug and Vaccine Development, National Institute of Infectious Diseases, Tokyo, Japan; * These authors contributed equally to this work and share first authorship.

**Keywords:** influenza, antiviral, resistance, oseltamivir, peramivir, baloxavir

## Abstract

An outbreak of influenza A(H1N1)pdm09 viruses exhibiting cross-resistance to oseltamivir and peramivir occurred in Yokohama, Japan, in September 2024. Among 24 students in a class, 11 were diagnosed with influenza or influenza-like illness, and viruses harbouring the NA H275Y and HA Q210H substitutions were isolated from four. Deep sequencing analysis confirmed the clonal spread of these mutants. Antigenic analysis revealed differences from the vaccine strain. Continued monitoring is crucial to assess the potential for further spread of these mutant viruses.

During nationwide monitoring of antiviral susceptibilities of influenza viruses in Japan, we detected 25 A(H1N1)pdm09 viruses in September 2024. Four of these viruses possessed the H275Y substitution in their neuraminidase (NA) protein, which is associated with cross-resistance to the NA inhibitors oseltamivir and peramivir [1]. These viruses were identified in students from the same class at a school. Here, we report an outbreak of the NA H275Y mutant A(H1N1)pdm09 virus in this school.

## An outbreak of influenza A(H1N1)pdm09 virus in a school

In Japan, local outbreaks of influenza have been reported since week 29 (starting on 15 July) of 2024 (FluNet, https://www.who.int/tools/flunet). One such outbreak occurred in a class of 24 students at a school in Yokohama, where 11 students aged between 6 and 7 years were absent from school on a day at the beginning of September 2024 (hereafter referred to as day 3) upon diagnosis with influenza or influenza-like illness. The class was subsequently closed for the next 3 days. The patients exhibited a fever over 38 °C, headache, and fatigue, but no severe illness was reported. On day 3, nasal discharges and gargle samples were collected from four of the 11 patients, and influenza A(H1N1)pdm09 viruses were isolated (Table 1). Of these four patients, two had developed symptoms 2 days before (day 1), and the other two on the previous day (day 2). One patient began oseltamivir treatment on the day of symptom onset, while the other three received oseltamivir or laninamivir on the day of sample collection (Table 1).

**Table 1:** Influenza A(H1N1)pdm09 viruses detected during an outbreak in a school in Yokohama, Japan, September 2024 (n = 4 cases)

GISAID isolate ID	Isolate name	Onset of symptoms	Specimen collection^a^	Antiviral treatment	NA substitution
19484762	A/Yokohama/34/2024	Day 1 2024	Day 3 2024	Day 3 2024 Oseltamivir started	H275Y
19484764	A/Yokohama/35/2024	Day 2 2024	Day 3 2024	Day 3 2024 Laninamivir	H275Y
19484766	A/Yokohama/36/2024	Day 2 2024	Day 3 2024	Day2 2024 Oseltamivir started	H275Y
19484768	A/Yokohama/37/2024	Day 1 2024	Day 3 2024	Day 3 2024 Oseltamivir started	H275Y

GISAID: Global Initiative on Sharing All Influenza Data; ID: identity; NA: neuraminidase.

^a^ Nasal discharges and gargle samples.

## Detection of NA H275Y mutant influenza A(H1N1)pdm09 viruses during the school outbreak

Deep sequencing analysis revealed that all four A(H1N1)pdm09 viruses (A/Yokohama/34/2024, A/Yokohama/35/2024, A/Yokohama/36/2024, and A/Yokohama/37/2024) possessed the H275Y substitution in their NA protein, which is associated with cross-resistance to oseltamivir and peramivir [1] (Table 1). Oseltamivir binds to the active site of NA in a similar manner to peramivir; therefore, the NA H275Y substitution alters the active site structure and confers resistance to both drugs. This substitution was confirmed in both the virus isolates and the clinical specimens. No amino acid substitutions associated with reduced susceptibility to the cap-dependent endonuclease inhibitor baloxavir were detected in the NA H275Y mutant viruses. The whole genome sequences of the viruses in the specimens were identical, demonstrating clonal spread of the NA H275Y mutant virus strain. These findings suggest an outbreak of the NA H275Y mutant virus within the school class.

## Antiviral susceptibility of the NA H275Y mutant influenza A(H1N1)pdm09 viruses

We assessed the susceptibilities of the NA H275Y mutant viruses to NA inhibitors (oseltamivir, peramivir, zanamivir, and laninamivir) and baloxavir by using a fluorescence-based NA inhibition assay and a focus reduction assay, respectively, as previously described [2]. Our results are expressed as 50% inhibitory concentration (IC_50_) values. Antiviral susceptibility was interpreted according to the World Health Organization (WHO) criteria, which are based on fold-changes in IC_50_ values compared with the median value for viruses of the same type/subtype/lineage [3]. For NA inhibitors, the criteria define normal (< 10-fold increase), reduced (10–100-fold increase), or highly reduced (> 100-fold increase) inhibition for influenza A viruses [4]. For baloxavir, the provisional criteria define influenza virus susceptibility as normal (≤ 3-fold increase) or reduced (> 3-fold increase) [3].

As shown in Table 2, the NA H275Y mutant viruses exhibited IC_50_ values 1,160- to 1,340-fold higher for oseltamivir, 203- to 254-fold higher for peramivir, 1.2- to 1.3-fold higher for zanamivir, 1.6- to 1.7-fold higher for laninamivir, and 0.9- to 1.2-fold higher for baloxavir, respectively, compared with the median IC_50_ values of wild-type A(H1N1)pdm09 viruses isolated in the 2024/25 influenza season in Japan. These results indicate that the NA H275Y mutant viruses from the outbreak show highly reduced inhibition by oseltamivir and peramivir but are susceptible to zanamivir, laninamivir, and baloxavir.

**Table 2:** Antiviral susceptibilities of influenza A(H1N1)pdm09 viruses detected in Japan, September 2024 (n = 7 virus isolates)

Substitution		Isolate name	IC_50_ (nM)				
NA	HA		NA inhibitors^a^ (WHO criteria^b^)				Baloxavir^c^ (fold-change^d^)
			Oseltamivir	Peramivir	Zanamivir	Laninamivir	
H275Y	Q210H	A/Yokohama/34/2024	268.28 (HRI)	25.01 (HRI)	0.49 (NI)	1.06 (NI)	5.97 (0.9)
H275Y	Q210H	A/Yokohama/35/2024	281.30 (HRI)	25.35 (HRI)	0.48 (NI)	0.99 (NI)	7.86 (1.2)
H275Y	Q210H	A/Yokohama/36/2024	260.35 (HRI)	22.37 (HRI)	0.50 (NI)	1.03 (NI)	7.00 (1.1)
H275Y	Q210H	A/Yokohama/37/2024	244.09 (HRI)	20.34 (HRI)	0.52 (NI)	0.95 (NI)	6.45 (1.0)
None	210Q	A/Yokohama/40/2024^e^	0.17 (NI)	0.10 (NI)	0.43 (NI)	0.65 (NI)	6.53 (1.0)
None	210Q	A/Kanagawa/IC2401/2024^e^	0.23 (NI)	0.09 (NI)	0.32 (NI)	0.48 (NI)	4.66 (0.7)
None	210Q	A/Kanagawa/IC2402/2024^e^	0.22 (NI)	0.10 (NI)	0.39 (NI)	0.58 (NI)	8.06 (1.2)
None	210Q	A(H1N1)pdm09 viruses in the 2024/25 season in Japan^f^	0.21 ± 0.03	0.10 ± 0.01	0.40 ± 0.05	0.61 ± 0.08	6.53 ± 1.16

HA: haemagglutinin; HRI: highly reduced inhibition; IC_50_: 50% inhibitory concentration; NA: neuraminidase; NI: normal inhibition; WHO: World Health Organization.

^a^ Mean IC_50_ values of duplicate reactions were determined by use of a fluorescence-based neuraminidase inhibition assay.

^b^ The WHO criteria define influenza A virus inhibition as normal (< 10-fold increase), reduced (10–100-fold increase), or highly reduced (> 100-fold increase).

^c^ Mean IC_50_ values of triplicate reactions were determined by use of a focus reduction assay.

^d^ Fold-change in IC_50_ values compared with median IC_50_ values. According to the WHO provisional criteria, a fold-change value > 3-fold is considered reduced susceptibility to baloxavir.

^e^ This is an A(H1N1)pdm09 virus isolated in Japan in September 2024.

^f^ Median IC_50_ values ± standard deviation of influenza A(H1N1)pdm09 viruses lacking amino acid substitutions associated with reduced susceptibility to antivirals isolated during the 2024/25 influenza season in Japan are displayed in this row of the table.

## Antigenicity of the NA H275Y mutant influenza A(H1N1)pdm09 viruses

The NA H275Y mutant viruses from the outbreak possessed the Q210H substitution in their haemagglutinin (HA) protein (Table 2). Analysis of A(H1N1)pdm09 virus HA sequences in the GISAID EpiFlu database revealed that this substitution has been sporadically detected worldwide but has not been identified in any of the 3,871 sequences of A(H1N1)pdm09 viruses isolated in Japan. The A(H1N1)pdm09 HA contains five major antigenic sites (Sa, Sb, Ca1, Ca2, and Cb) [5], with position 210 forming part of the Sb antigenic site [6].

We evaluated the antigenicity of two NA H275Y mutant viruses (A/Yokohama/36/2024 and A/Yokohama/37/2024) since the genetic sequences of all four mutant viruses were identical. This evaluation was performed using a focus reduction neutralisation test (FRNT) with anti-HA monoclonal antibodies against A/Narita/1/2009(H1N1)pdm09, as previously reported [6] (Table 3). Viruses were considered antigenically distinct when their 50% focus reduction neutralisation titre (FRNT_50_) values showed a > 4-fold difference relative to the vaccine strain [7], as previously described [8].

**Table 3:** Antigenicity of influenza A(H1N1)pdm09 viruses, including two detected during a school outbreak in Yokohama, Japan, September 2024 (n = 5 virus isolates)

Virus isolates	HA clade/subclade	HA substitution	NA substitution	FRNT_50_^a^							
				Sa^b^					Sb^b^	Ca2^b^	
				n9^c^	n10^c^	n11^c^	n12^c^	n13^c^	n16^c^	n17^c^	n18^c^
**Reference viruses**											
A/Narita/1/2009	6B	210Q	275H	89,378	37,432	20,412	242,918	12,709	19,970	177,038	50,794
A/Wisconsin/67/2022^d^	6B.1A.5a.2a.1/C.1.1	210Q	275H	520	95	416	1,265	153	39	345	975
**Test viruses**											
A/Kanagawa/IC2401/2024^e^	6B.1A.5a.2a/C.1.9	210Q	275H	202	43	395	579	108	30	173	**165**
A/Yokohama/36/2024	6B.1A.5a.2a/C.1.9	Q210H	H275Y	2,072	**421**	223	**7,633**	186	< 20	115	**149**
A/Yokohama/37/2024	6B.1A.5a.2a/C.1.9	Q210H	H275Y	1,520	**628**	346	**5,812**	176	< 20	116	**137**

FRNT_50_: 50% focus reduction neutralisation titre; HA: haemagglutinin; NA: neuraminidase.

^a^ FRNT_50_ values of duplicate reactions were determined by use of a focus reduction neutralisation test with anti-HA monoclonal antibodies against A/Narita/1/2009, an early strain isolated in Japan. FRNT_50_ values that show a > 4-fold difference relative to the vaccine strain A/Wisconsin/67/2022 are in bold.

^b^ Antigenic sites of HA.

^c^ Anti-HA monoclonal antibody against A/Narita/1/2009(H1N1)pdm09.

^d^ A vaccine strain recommended by the World Health Organization for cell culture-based vaccine for the 2024/25 northern hemisphere influenza season.

^e^ A representative A(H1N1)pdm09 virus isolated in Japan, September 2024.

Interpreting the results of the n16 antibody, which recognises the Sb site including position 210, was challenging due to its low reactivity to the vaccine strain recommended by the WHO for the 2024/25 northern hemisphere influenza season, A/Wisconsin/67/2022. A representative wild-type virus recently circulating in Japan (A/Kanagawa/IC2401/2024) and the NA H275Y mutant viruses exhibited reduced reactivity to the n18 antibody, which targets the Ca2 site. However, the reactivity of A/Kanagawa/IC2401/2024 to other antibodies was similar to that of the vaccine strain. In contrast, the NA H275Y mutant viruses showed a > 4-fold difference in reactivity to the n10 and n12 antibodies and a notable difference in reactivity to the n9 antibody, all of which recognise the Sa site, which is contiguous with the Sb site and suggested to share a close linkage [6]. These results indicate that the antigenicity of the NA H275Y mutant viruses, which possess the HA Q210H substitution, differs from that of the vaccine strain recommended for the 2024/25 northern hemisphere season, whereas the antigenicity of a recently circulating virus that does not possess the HA Q210H substitution is relatively similar to that of the vaccine strain.

## Growth capability of the NA H275Y mutant influenza A(H1N1)pdm09 viruses

To evaluate the impact of the NA H275Y substitution on viral growth in vitro, humanised Madin-Darby canine kidney (MDCK) cells (i.e. hCK cells) [9] were infected with either a representative NA H275Y mutant virus (A/Yokohama/36/2024) or a recently circulating wild-type virus (A/Kanagawa/IC2401/2024) at a multiplicity of infection of 0.001 focus-forming units per cell. Supernatants were collected at different time points and virus titres were determined by using a focus assay, as illustrated in the Figure. The growth kinetics of the NA H275Y mutant virus showed reduced replication compared with the wild-type virus during the early time points post-infection. However, both the mutant and wild-type viruses reached similar titres at later time points, indicating that the NA H275Y substitution does not significantly impair the overall replication capacity of the A(H1N1)pdm09 virus at least in vitro.

**Figure:**
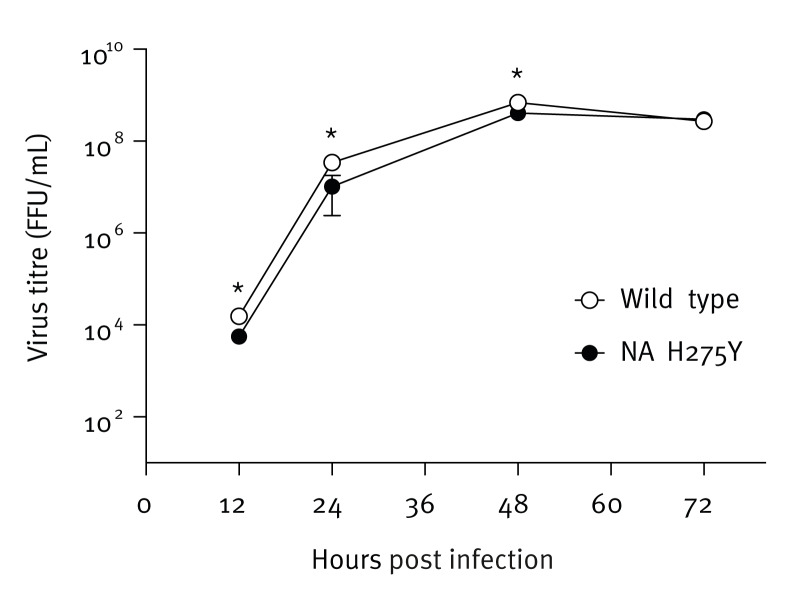
In vitro growth kinetics of an NA H275Y mutant influenza A(H1N1)pdm09 virus compared to a wild type influenza A(H1N1)pdm09 virus from the same year, Japan, September 2024 (n = 2 virus strains)

FFU: focus-forming units; MDCK: Madin-Darby canine kidney; NA: neuraminidase.

Humanised MDCK cells (i.e. hCK cells) were infected with NA H275Y mutant virus (A/Yokohama/36/2024) or wild-type virus (A/Kanagawa/IC2401/2024) at a multiplicity of infection of 0.001 FFU per cell in triplicate. Supernatants were harvested at the indicated time points and subjected to virus titration by using a focus assay. Means (represented as filled or non-filled circles) and standard deviations (represented by whiskers) are shown. Asterisks indicate statistically significant differences between the mutant and wild-type viruses as determined by using a t-test calculated via a mixed-effects model (*p < 0.05).

## Discussion

In this study, an outbreak of influenza A(H1N1)pdm09 antigenic variants exhibiting cross-resistance to oseltamivir and peramivir was detected on a day in September 2024 (day 3) in a class at a school in Yokohama, Japan. Three days later (day 6), at the same school, five of 22 students aged between 10 and 11 years in another class were absent due to diagnosis with influenza or influenza-like illness. Closure of the class was implemented from day 7 to day 9 but the pathogen responsible for these five students’ symptoms was not investigated.

In Japan, anti-influenza drugs are commonly prescribed to outpatients without underlying disease [10]. During the 2013/14 season, a large community cluster of NA H275Y mutant A(H1N1)pdm09 viruses occurred in Hokkaido, Japan [11]. The detection rate of these mutant viruses reached 29% in Hokkaido during that season; however, they were eventually replaced by wild-type virus and disappeared [12]. Our previous findings suggest that the cluster virus retained sufficient viral fitness to spread among humans, resulting in a large number of infections. However, the mutant NA structure was less stable than that of the wild-type virus, and once the wild-type virus began circulating in the community, the mutant virus could not compete and eventually disappeared [12]. Since then, the frequency of viruses with reduced susceptibility to NA inhibitors has remained low, less than 2% [3]. However, the growth capability of the oseltamivir- and peramivir-cross-resistant antigenic variants in this study, which possess the NA H275Y and HA Q210H substitutions, was comparable to that of the wild-type virus, at least at later time points in vitro. Future studies are needed to evaluate the viral fitness of these antigenic variants and to assess the biological significance of these substitutions. 

Our study has some limitations. While samples were collected from ill students of the class where the outbreak was first detected, clinical specimens were not obtained from five students who experienced influenza or influenza-like illness 3 days later in another class of the same school; therefore, virus analysis could not be conducted, and the potential for further spread of the viruses implicated in the outbreak could not be assessed.

## Conclusion

In this outbreak of influenza A(H1N1)pdm09, which affected a Japanese school in September 2024, analyses of viruses affecting four cases revealed cross-resistance to oseltamivir and peramivir, the presence of NA H275Y and HA Q210H mutations and evidence of clonal spread. The viruses moreover exhibited antigenic differences to the seasonal vaccine strain. With their growth being comparable to that of wild-type A(H1N1)pdm09 virus in vitro, at least at later time points, continued monitoring is crucial to assess the potential for further spread of these variants.
